# Spatial profiling of the metabolism-immune axis in ovarian cancer

**DOI:** 10.3389/fphar.2025.1672020

**Published:** 2026-01-29

**Authors:** Zhi-Bin Wang, Ming-Hui Long, Ping Yu, Ya-Li Wang, Zheng Yang, Ma-Sha Huang

**Affiliations:** 1 Department of Pharmacy, The Affiliated Cancer Hospital of Xiangya School of Medicine, Central South University/ Hunan Cancer Hospital, Changsha, China; 2 Department of Pharmacy, Ruijin Hospital, Shanghai Jiao Tong University School of Medicine, Shanghai, China; 3 Department of Physiology and Pathophysiology, Henan Medical University, Xinxiang, China; 4 Department of Biochemistry and Molecular Cell Biology, Shanghai Key Laboratory for Tumor Microenvironment and Inflammation, Shanghai Jiao Tong University, School of Medicine, Shanghai, China

**Keywords:** immunometabolic crosstalk, metabolic reprogramming, ovarian cancer, spatial multi-omics, tumor microenvironment

## Abstract

Ovarian cancer remains a lethal disease marked by profound therapeutic resistance, largely orchestrated by a complex tumor microenvironment (TME) governed by metabolism-immune crosstalk. This review focuses on the spatiotemporal dynamics of the metabolism-immune axis in ovarian cancer progression and resistance, with particular emphasis on how cutting-edge spatial multi-omics technologies reveal previously unrecognized layers of intratumoral heterogeneity and geographic organization that cannot be captured by bulk analyses. Using tools such as MALDI-MSI, GeoMx DSP, and CODEX, these approaches enable high-resolution, spatially resolved mapping of metabolite gradients (e.g., lactate, lipids, kynurenine), immune cell niches, and immunometabolic checkpoints within distinct tumor regions. Such spatial profiling uncovers how metabolic reprogramming-dysregulated glycolysis, lipid metabolism, and glutaminolysis-drives localized immunosuppression and chemoresistance through compartment-specific interactions among tumor cells, cancer-associated fibroblasts (CAFs), adipocytes, and immune populations. These geographically defined insights reshape our understanding of therapeutic failure and highlight precise, location-aware vulnerabilities. Accordingly, we propose spatially informed therapeutic strategies, including regional glycolysis inhibition, glutaminase blockade, lipid pathway interference, and their rational combination with immune checkpoint inhibitors (ICIs), to disrupt pathogenic metabolic-immune circuits and improve immunotherapy outcomes. Looking ahead, advances in vivo spatial imaging, gut microbiota modulation, and AI-powered integrative multi-omics frameworks promise truly personalized treatment of ovarian cancer.

## 1 Introduction

Ovarian cancer exhibits the highest mortality rate among gynecologic malignancies. Due to nonspecific symptoms, approximately 70% of cases are diagnosed at advanced stages, contributing to poor overall prognosis (Cortez et al., 2018; Colombo et al., 2019). The high lethality and therapeutic challenges stem from profound tumor heterogeneity, a characteristic peritoneal dissemination pattern, and microenvironment-driven drug resistance. Conventional therapeutic paradigms face limitations due to their oversight of dynamic spatiotemporal interactions between tumor and stroma and the evolving metabolism-immune network (Cheng et al., 2009; Hansen et al., 2016; Worzfeld et al., 2017; Kossaï et al., 2018). The current standard-of-care involves cytoreductive surgery combined with platinum-based chemotherapy (Veneziani et al., 2023); however, microscopic residual disease frequently persists post-surgery (Narod, 2016). Following initial platinum therapy, approximately 85% of patients with epithelial ovarian cancer experience recurrence, often progressing to chemoresistant disease (St Laurent and Liu, 2024). Furthermore, PARP inhibitors demonstrate efficacy primarily within the homologous recombination deficiency (HRD)-positive subset (Kaye, 2016). Critically, conventional strategies also neglect the immunosuppressive microenvironment orchestrated by components such as cancer-associated fibroblasts (CAFs) and tumor-associated macrophages (TAMs). Chemotherapy itself can paradoxically increase PD-L1 expression, fostering a therapy-induced remodeling of the microenvironment that contributes to immunotherapy failure (Jiménez-Sánchez et al., 2020). As a disease encompassing diverse molecular subtypes, the inherent heterogeneity of ovarian cancer results in variable degrees of intrinsic resistance to conventional platinum chemotherapy. This heterogeneity further drives subtype-specific alterations in the TME, adaptive immune tolerance, and therapeutic susceptibility (Veneziani et al., 2023). Despite this complexity, clinical management remains largely guided by uniform histopathological classification. Consequently, targeted agents directed against aberrations like PI3K/AKT pathway dysregulation or HRD exhibit suboptimal efficacy when applied without consideration of the specific molecular context within ovarian cancer subtypes (Tse et al., 2014).

The dynamic TME ecosystem serves as a central hub driving therapeutic resistance. Comprising heterogeneous cell populations, extracellular matrix, and chemokines, the ovarian cancer TME features core components such as immunosuppressive cells, CAFs, adipocytes, and nascent vascular networks. These elements interact through complex mechanisms to drive immune evasion, chemoresistance, and metastasis (Chap et al., 2024). Infiltrating regulatory T cells (Tregs), M2-polarized TAMs, and myeloid-derived suppressor cells (MDSCs) establish an immunosuppressive barrier by secreting cytokines like IL-10 and TGF-β to inhibit effector T cell function while concurrently inducing immune checkpoint molecules such as PD-1/PD-L1 (Zhu and Paul, 2010; Maimon et al., 2021; Sun et al., 2024; Kumagai et al., 2022). CAFs promote angiogenesis, provide tumor support, enhance cancer cell survival, and critically modulate T cell state and localization within solid tumors (Arpinati et al., 2024). Cancer-associated adipocytes further contribute by secreting adipokines, cytokines, and growth factors that disrupt endocrine signaling to tumor cells (Sabol et al., 2019). Collectively, this intricate signaling crosstalk among diverse TME cell populations drives cancer stem cell maintenance (Nallasamy et al., 2022). Consequently, traditional platinum-based, “one-size-fits-all” therapeutic strategies often fail to achieve desired efficacy due to their disregard for the TME’s spatiotemporal heterogeneity. Physical barriers formed by CAFs impede drug penetration, the immunosuppressive milieu attenuates immunotherapy response, and metabolic reprogramming undermines targeted therapies, culminating in an inability to meet personalized patient needs.

A profound understanding of the spatiotemporal relationships governing the metabolic-immune network is crucial for overcoming therapeutic bottlenecks. The dynamic interplay between metabolism and immunity within the TME significantly influences treatment response and clinical outcomes. While foundational studies established that tumor cells gain proliferative advantage through metabolic reprogramming strategies such as the Warburg effect and glutamine addiction (Koppenol et al., 2011; Altman et al., 2016), recent insights reveal that this metabolic rewiring extends beyond self-sufficiency. Tumor cells actively reshape the immune microenvironment via metabolite exchange, nutrient competition, and signaling crosstalk (Zhong et al., 2022; Morandi et al., 2016). Conversely, aberrant microenvironmental conditions-including hypoxia, low pH, and nutrient deprivation-trigger adaptive responses in tumor cells, such as autophagy, which further modulate cellular metabolism (Cairns et al., 2011). However, conventional research, often limited to *in vitro* metabolomic analysis at the cellular level, overlooks the three-dimensional spatial compartmentalization of metabolic gradients and intercellular communication between tumor parenchyma and stroma. This review aims to address this limitation by integrating a spatial multi-omics perspective. We focus specifically on spatial transcriptomics, proteomics, and metabolomics technologies, as they enable decoding of the functional spatial network of the metabolic-immune axis across the complete chain from gene expression and protein function to metabolite-mediated cell crosstalk-dynamic processes that cannot be captured by genomic analysis alone. Therefore, integrating spatial metabolomics, single-cell transcriptomics, and multiplexed fluorescence imaging to systematically delineate the spatiotemporal coupling of metabolic enzyme localization, metabolite diffusion boundaries, and immune cell functional states will provide a crucial theoretical foundation. This approach will inform the development of precision interventions targeting metabolic-immune crosstalk, potentially reversing the immunosuppressive TME by alleviating nutrient deprivation and reprogramming the metabolic adaptability of immune cells.

## 2 Metabolic reprogramming in ovarian cancer: from molecular mechanisms to spatial heterogeneity

### 2.1 Core hallmarks of metabolic reprogramming

Metabolic reprogramming constitutes a critical driver of malignant progression and therapy resistance in ovarian cancer. Its defining features encompass spatiotemporally dysregulated pathways governing glucose, lipid, and amino acid metabolism. While conventional research has predominantly focused on molecular mechanisms within isolated metabolic pathways, it has largely overlooked the spatial organization (‘spatial heterogeneity’ specifically refers to the diverse distribution patterns of molecular, cellular, and metabolic characteristics across different spatial regions of the TME) of metabolic heterogeneity within the TME. This section systematically dissects the regulatory networks underpinning glycolysis, lipid metabolism, and glutamine dependence. By integrating spatial omics technologies, we delineate the three-dimensional heterogeneity inherent to metabolic reprogramming and elucidate the cooperative oncogenic mechanisms arising from the spatial localization of metabolic enzymes and their interplay with the TME. Collectively, this analysis establishes a theoretical foundation for spatially precise interventions targeting the metabolism-immune axis.

Our dscussion of glycolytic reprogramming centers on key regulatory nodes-HK2, lactate dehydrogenase A (LDHA), and monocarboxylate transporters (MCTs)-based on their established and irreplaceable roles in driving ovarian cancer progression and shaping the immune microenvironment. HK2 is highlighted over other hexokinase isoforms due to its prominent overexpression in cancer, the anti-apoptotic benefit afforded by its mitochondrial localization, and its direct involvement in initiating glycolysis-positioning it as the gatekeeper of glucose entry (Hu et al., 2014). LDHA is underscored as the critical enzyme that converts pyruvate to lactate, directly coupling glycolytic flux to the production of the immunosuppressive metabolite lactate. Its activity is indispensable for maintaining the NAD+/NADH balance necessary for sustained glycolytic activity (Zhang et al., 2022; Sharma et al., 2025). MCTs (particularly MCT1 and MCT4) are highlighted as the primary channels for lactate efflux (from tumor cells) and influx (into stromal or immune cells). Their spatial expression patterns underpin the establishment of a lactate-enriched acidic niche, a hallmark of the immunosuppressive microenvironment. Targeting these transporters disrupts the entire “lactate shuttle” system (Ibacache‐Chía et al., 2024; Hatami et al., 2023). This focus on HK2, LDHA, and MCTs enables a mechanistic dissection of how glycolytic flux is initiated, executed, and spatially communicated within the TME, rather than providing a comprehensive but superficial catalog of all glycolytic enzymes.

**FIGURE 1:**
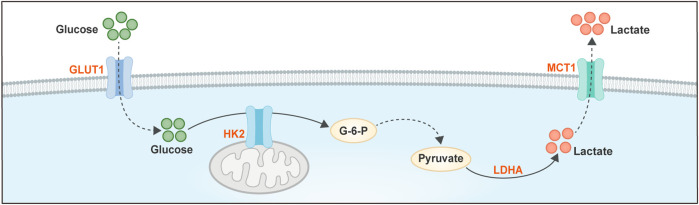
Dynamic Coordination of Aberrant Glucose Metabolism and Lactate Shuttling in Ovarian Cancer. HK2, localized on the outer mitochondrial membrane, phosphorylates glucose to accelerate glycolysis. This process occurs even under aerobic conditions, a phenomenon known as the Warburg effect, wherein cancer cells preferentially rely on glycolysis. Therein, LDHA catalyzes the conversion of the resulting pyruvate to lactate, thereby regenerating NAD^+^ to sustain glycolytic flux. Subsequently, the exported lactate is shuttled out of the cell via MCTs. This acidifies the tumor microenvironment, ultimately promoting tumor invasion and immune evasion. HK2, Hexokinase 2; LDHA, Lactate dehydrogenase A; MCTs, monocarboxylate transporters.

In ovarian cancer, glycolytic reprogramming exhibits unique clinical correlations. Malignant ascites from high-grade serous ovarian cancer (HGSOC) contains elevated levels of lactate, which is significantly associated with chemoresistance and poor prognosis (Mukherjee et al., 2015; Rashid et al., 2025). Studies have demonstrated that the expression of HK2 and LDHA in ovarian cancer cell lines (e.g., SKOV3, OVCAR3) is directly linked to paclitaxel resistance, while inhibition of LDHA restores chemosensitivity. Furthermore, single-cell RNA sequencing (scRNA-seq) has revealed marked intratumoral heterogeneity in glycolytic activity, with cell subsets displaying strong epithelial-mesenchymal transition (EMT) signatures showing the most robust enrichment of glycolytic pathways (Xiao et al., 2020). Aberrant glucose metabolism, exhibiting pronounced spatial heterogeneity, represents a fundamental characteristic of metabolic reprogramming in ovarian cancer. This dysregulation involves dynamic coordination of the Warburg effect and lactate shuttling (Figure 1). Key enzymes, HK2 and LDHA, act synergistically to drive malignant progression (Hirschhaeuser et al., 2011; Wang and Patti, 2023). HK2, localized to the outer mitochondrial membrane, phosphorylates glucose to glucose-6-phosphate. This reaction not only accelerates glycolysis but also suppresses mitochondrial apoptosis signaling by modulating the mitochondrial permeability transition pore (mPTP), as evidenced in mouse ischemia-reperfusion models, conferring a survival advantage upon tumor cells (Mathupala et al., 2009; Andrienko et al., 2017). Conversely, LDHA catalyzes the conversion of pyruvate to lactate, facilitating NAD^+^ regeneration to sustain glycolysis. Crucially, lactate efflux via MCTs acidifies the extracellular milieu, thereby promoting tumor invasion and immune evasion (Song et al., 2020; Sharma et al., 2022). Spatial metabolomics, particularly mass spectrometry imaging (MSI)-based approaches, offers unique advantages by enabling direct, label-free, and molecularly specific detection of a broad range of metabolites at (sub)micrometer resolution, thereby directly visualizing how metabolic activities are spatially regulated. Furthermore, spatial analysis of candidate drug metabolic responses enables the early identification of detrimental metabolic effects during drug development (Alexandrov, 2023). Applying spatial metabolomics to ovarian cancer research holds transformative potential: it may overcome therapy resistance stemming from metabolic adaptability and facilitate the evaluation of novel therapeutic agents.

Although we have emphasized the synergistic roles of HK2 and LDHA due to their direct links to lactate production and immunosuppression, glycolytic flux is tightly regulated at three irreversible steps. Beyond HK2, PFK-1 and pyruvate kinase (PK) are equally critical nodes. Their regulatory isoforms-PFKFB3 and PKM2-exhibit distinct spatial distribution patterns within the TME, dictating metabolic plasticity and anabolic support.

PFK-1 and PFKFB3 serve as the primary flux-controlling enzymes in glycolysis. PFK-1 activity is potently regulated by PFKFB3, which synthesizes fructose-2,6-bisphosphate (F2,6BP)-the most effective allosteric activator of PFK-1. PFKFB3 is frequently upregulated in cancer and displays spatially heterogeneous expression patterns, often enriched in regions of hypoxia and nutrient stress. Its high activity drives a “glycolytic burst,” diverting carbon flux into the pathway to support biomass synthesis (Yang et al., 2025). Spatial transcriptomics may reveal PFKFB3-high expression niches adjacent to proliferating tumor cells, while its relative absence may mark more quiescent or oxidative regions (Ha et al., 2018). PKM2 is a well-recognized cancer hallmark. Its low catalytic activity and ability to exist as a dimer (in contrast to the highly active tetrameric PKM1) create a metabolic “bottleneck,” allowing upstream glycolytic intermediates to accumulate and be shunted into biosynthetic pathways (e.g., the pentose phosphate pathway for nucleotide synthesis) (Mukherjee et al., 2015; Wa et al., 2023). Critically, the spatial localization and oligomeric state of PKM2 are likely heterogeneous. At the invasive front, it may predominantly exist as a nuclear dimer, functioning as a protein kinase to promote gene expression; in the tumor core, it may be primarily in the metabolically active tetrameric form (Wa et al., 2023). This spatial regulation directly links glycolytic flux to anabolic growth and invasive programs. In summary, the glycolytic landscape of the ovarian cancer TME is collectively shaped by the spatial co-expression and activity of HK2, PFKFB3, and PKM2 (Zhang et al., 2020). HK2 controls entry into the pathway, PFKFB3 governs flux rate, and PKM2 determines the fate of carbon flux-whether toward lactate production or biomass synthesis. This expanded perspective reveals a more complex and hierarchical metabolic control system that future spatial metabolomics studies must dissect to fully understand and target glycolysis in ovarian cancer (Zhang et al., 2022; Wa et al., 2023).

Ovarian cancer displays tissue-specificity in lipid metabolic reprogramming. Endometriosis-associated clear cell ovarian carcinoma (CCOC) is characterized by a distinct lipid accumulation phenotype, whereas high-grade serous ovarian carcinoma (HGSOC) is more dependent on fatty acid oxidation (FAO). Preclinical studies have demonstrated that ovarian cancer stem cells (CSCs) overexpress the fatty acid-binding protein FABP4, sustaining their stemness via enhanced fatty acid uptake (Ha et al., 2018). Critically, LPA is present at abnormally high levels in ovarian cancer ascites, which directly drives tumor progression and immunosuppression through activation of the LPA receptor-STAT3 signaling cascade (Mukherjee et al., 2015; Ha et al., 2018). Within the TME, a core regulatory network centered on lipid metabolic reprogramming maintains dynamic equilibrium by integrating fatty acid uptake, synthesis, and oxidation pathways, thereby driving malignant progression (Röhrig and Schulze, 2016; Jin H. R. et al., 2023). Fatty acid translocase CD36 facilitates the uptake of exogenous lipids by tumor cells from the microenvironment, while fatty acid synthase (FASN) catalyzes the *de novo* synthesis of endogenous long-chain fatty acids. Together, these processes fulfill the heightened membrane biogenesis demands of rapidly proliferating tumors (Hao et al., 2020; Menendez and Lupu, 2007). Concurrently, CPT1A, the rate-limiting enzyme for mitochondrial β-oxidation, sustains tumor cell survival under nutrient deprivation by regulating FAO to generate energy (Ma L. et al., 2024). Significantly, tumor cells and stromal components-such as CAFs and adipocytes-establish a bidirectional lipid exchange network via exosomes, lipid transfer proteins, and metabolic paracrine signaling. Stromal cells secrete free fatty acids or lipid droplets for tumor cell uptake, while tumor cells reciprocally modulate stromal lipolysis through the release of signaling molecules like lactate and reactive oxygen species (ROS), fostering a pro-tumorigenic metabolic symbiosis (Jin H. R. et al., 2023; Hoy et al., 2021). The application of spatial lipidomics offers a powerful strategy to spatially track distinct lipid species within ovarian cancer. This approach can elucidate how lipid-based TME interactions drive disease progression and therapy resistance, potentially enabling early detection or facilitating large-scale screening of high-risk populations for preventive strategies (Wang G. et al., 2022; Mirretta Barone et al., 2024).

Glutamine metabolism exhibits subtype-specific features in ovarian cancer. Clear CCOC displays high sensitivity to glutamine deprivation, which correlates with the expression level of ASCT2 (SLC1A5). In contrast, high-grade serous ovarian cancer (HGSOC) utilizes glutamine to support the tricarboxylic acid (TCA) cycle through anaplerotic reactions. Studies have shown that the glutaminase inhibitor CB-839 exerts a synergistic anti-tumor effect in BRCA-mutant ovarian cancer models, suggesting that the HRD status may influence glutamine metabolic dependency (Vikramdeo et al., 2025; Hanahan et al., 2025). Therapeutic resistance is closely linked to the metabolic adaptability of tumor cells, with glutamine dependence and amino acid metabolic plasticity serving as key regulatory mechanisms. The primary glutamine transporter ASCT2 (SLC1A5), in concert with GLS1, drives glutaminolysis. This pathway generates α-ketoglutarate (α-KG) to replenish the TCA cycle and maintain redox balance (Du et al., 2022). Studies reveal that chemoresistant tumor cells upregulate ASCT2 to enhance glutamine uptake and utilization, subsequently activating the mTORC1 signaling pathway and promoting nucleotide biosynthesis. This adaptive response mitigates metabolic stress induced by chemotherapeutic agents (Wang et al., 2013). Integrated proteomic and metabolomic analyses have delineated two distinct ovarian cancer subtypes: a low-oxidative phosphorylation (OXPHOS) subtype reliant on glycolysis and a high-OXPHOS subtype. The high-OXPHOS subtype exhibits increased sensitivity to conventional chemotherapy, attributed to its augmented fatty acid oxidation capacity (Gentric et al., 2019). Leveraging single-cell metabolomics holds promise for uncovering latent metabolic subtypes within ovarian cancer and characterizing their specific metabolic dependencies. Combining glutaminolysis or fatty acid oxidation inhibitors with conventional chemotherapeutics represents a potential strategy to overcome chemoresistance in this disease.

### 2.2 Interplay between metabolic reprogramming and an immunosuppressive microenvironment

Immunosuppression in the ovarian cancer microenvironment is intrinsically governed by metabolic alterations in malignant and stromal compartments. This bidirectional metabolic-immune crosstalk displays significant spatial heterogeneity, with localized enrichment of specific metabolites establishing and maintaining an immune-tolerant microenvironment through distinct molecular mechanisms. Understanding this dynamic provides key insights for deciphering immune evasion and designing spatially informed therapeutic strategies.

Lactate, a quintessential glycolytic byproduct, exemplifies how metabolites transcend metabolic signatures to actively construct immunosuppressive networks (Martinez-Outschoorn et al., 2017; Chen et al., 2025; Certo et al., 2021). Extracellular acidification by lactate impedes CD8^+^ T cell proliferation and cytotoxicity, an effect mediated through metabolic reprogramming associated with glycolytic activity (Kumagai et al., 2022). Furthermore, lactate dynamically calibrates the immunosuppressive function of Tregs based on microenvironmental metabolic cues. Spatial metabolomic profiling demonstrates that Tirzepatide (TZP), a dual GIP/GLP-1 receptor agonist, markedly depletes key glycolytic metabolites in colon cancer tissues, attenuating glycolytic flux. This underscores the role of pyruvate and lactate production in inducing tumor cell nutrient stress (Zhang Y. et al., 2025). Crucially, spatial metabolomics reveals strong histological concordance between lactate and glutamate metabolic zones. This spatial coupling implies a coordinated metabolic program: high glycolytic flux leading to lactate production coincides with increased glutaminolysis demand and may fuel the latter. Glutamate, a key product of glutamine metabolism, can enter the TCA cycle anaplerotically to sustain the production of biosynthetic precursors. Even in the context of truncated metabolism in the form of the Warburg effect (i.e., preferential anaerobic glycolysis), glutamate metabolism compensates for the TCA cycle to support metabolic flexibility (Li et al., 2023; Su et al., 2023). The colocalization of these metabolites highlights regions of intense metabolic activity and active nutrient recycling, which may serve to identify niches of aggressive tumor growth or adaptive resistance. Critically, spatial metabolomics reveals strong histological congruence between lactate and glutamate metabolic regions, and the distinct features of these metabolic niches facilitate the identification of areas associated with aggressive tumor growth or adaptive resistance (Li et al., 2023; de la Cruz-López et al., 2019; Gupta et al., 2017). Extensive regulatory interplay connects glutamine metabolism, lactate handling, glycolysis, and glutathione pathways (Zhu et al., 2023), highlighting the integrated nature of TME metabolism. This coordination reflects adaptive metabolic reprogramming, acidotic pressures, and cellular interactions, potentially unveiling novel targets for ovarian cancer therapy.

Lipid metabolic reprogramming constitutes another critical axis of immune microenvironment regulation, with metabolites dictating macrophage polarization states. AMPK-PPARα signaling activation by lipid species drives TAMs towards an M2 phenotype (Wang et al., 2025). These M2-polarized TAMs enforce immunosuppression via IL-10 and TGF-β secretion coupled with diminished MHC class II expression (Singhal et al., 2019). Polyunsaturated fatty acids (PUFAs) also facilitate M2-like TAM enrichment by suppressing RhoA-YAP1 signaling in ovarian cancer (Wang et al., 2024). Multiplex immunohistochemistry (mIHC) identified p53-deficient CSCs as the primary subset secreting IL-34. IL-34 upregulates CD36-dependent fatty acid oxidation, promoting the polarization of lipid-laden ‘foamy’ TAMs toward an M2 phenotype. These M2-type TAMs subsequently inhibit CD8^+^ T cell cytotoxicity through multifaceted crosstalk mechanisms. Key inhibitory effects include: (i) high expression of co-inhibitory ligands, such as PD-L1, which directly binds to the PD-1 receptor on T cells to deliver intrinsic inhibitory signals; (ii) robust secretion of immunosuppressive cytokines, such as IL-10 and TGF-β, which suppress T cell receptor signaling and effector gene expression; and (iii) creation of a metabolically adverse microenvironment by sequestering essential amino acids (e.g., L-arginine) and producing lactate, collectively impairing T cell metabolic fitness and proliferative capacity. This synergistic process effectively compromises CD8^+^ T cell immunity and facilitates immune evasion (Nian et al., 2024).

In amino acid metabolism, the tryptophan-kynurenine-AhR axis serves as a key regulator of immunosuppression in the ovarian cancer TME. Evidence from ovarian cancer models and patient samples shows elevated expression of IDO1/TDO2 and increased kynurenine levels, which correlate with enhanced Treg abundance and impaired CD8^+^ T cell function. This establishes kynurenine metabolism as a well-recognized component of the immunosuppressive network in ovarian cancer (Hanahan et al., 2025; de la Cruz-López et al., 2019). Within amino acid metabolism, the tryptophan metabolite kynurenine acts as a pivotal regulator, activating the aryl hydrocarbon receptor (AhR) signaling pathway to suppress effector T cell function and promote regulatory T cell (Treg) differentiation. This reveals an additional dimension of metabolic-immune crosstalk. Kynurenine inhibits effector T cell proliferation and enhances Treg generation through AhR activation (Cui et al., 2023; Mezrich et al., 2010; Uyttenhove et al., 2003). While direct spatial multi-omics evidence in ovarian cancer continues to emerge, pioneering studies in other immunologically “hot” and aggressive cancer types provide a compelling conceptual framework. For example, high-throughput digital spatial profiling conducted in TNBC-a malignancy that shares similarities with HGSOC, often exhibiting TP53 mutations, genomic instability, and an immunosuppressive TME-identified enrichment of the rate-limiting metabolic enzyme indoleamine 2,3-dioxygenase 1 (IDO1) in specific compartments, which correlates with patient prognosis. Notably, this study found that IDO1 is co-expressed with PD-L1 in early-stage TNBC, suggesting dynamic microenvironmental crosstalk between IDO1-driven tryptophan catabolism and the development of T cell exhaustion. This co-expression pattern of a key immunometabolic enzyme and a classical immune checkpoint provides critical multi-omics evidence for a conserved immune evasion mechanism highly relevant to ovarian cancer. It exemplifies how spatial technologies can uncover functional niches where metabolic and immunosuppressive pathways converge, offering a testable model for future validation in ovarian cancer specimens (Carter et al., 2023). Notably, IDO1 co-expresses with PD-L1 in early-stage TNBC, suggesting dynamic microenvironmental crosstalk between IDO1-driven tryptophan catabolism (producing kynurenine) and the development of T cell exhaustion phenotypes. This co-expression pattern of metabolic enzymes and immune checkpoints provides crucial multi-omics evidence for deciphering immune evasion mechanisms in ovarian cancer. Furthermore, this suggests that targeting the AhR-IDO1 axis may reverse immunosuppression by remodeling the metabolic landscape. This is supported by ovarian cancer studies, where kynurenine production and AhR activation have been mechanistically linked to the induction of Tregs and suppression of anti-tumor immunity, thereby opening new therapeutic avenues for combination immunotherapy in ovarian cancer (Amobi-McCloud et al., 2021).

**FIGURE 2:**
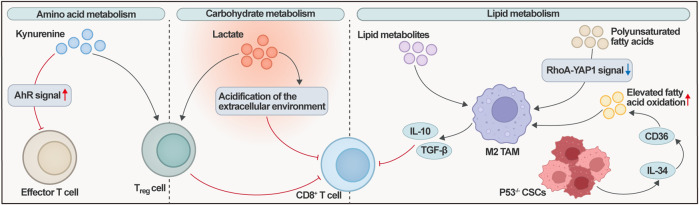
Spatial Interaction Network of the Metabolism-Immune Axis in Ovarian Cancer. Key metabolic pathways, including amino acid metabolism (kynurenine and AhR signaling), carbohydrate metabolism (lactate and extracellular acidification), and lipid metabolism (polyunsaturated fatty acids, CD36, and elevated fatty acid oxidation), are shown to influence immune cell populations. Effector T cells are suppressed by kynurenine-mediated AhR signaling. Lactate from carbohydrate metabolism acidifies the extracellular environment, inhibiting CD8^+^ T cell function and promoting Treg activity. Lipid metabolism drives M2 TAM polarization through RhoA-YAP1 signaling and IL-34 secretion by p53−/− CSCs, further enhancing immunosuppression via IL-10 and TGF-β. This spatial network underscores the dynamic crosstalk driving immune evasion and therapeutic resistance. AhR, Aryl Hydrocarbon Receptor; PUFAs, Polyunsaturated Fatty Acids; Tregs, Regulatory T cells; CSCs, Cancer Stem Cells; TAMs, Tumor-Associated Macrophages.

In summary, metabolic reprogramming within spatially defined ovarian cancer microenvironments-encompassing dysregulated glycolysis, lipid metabolism, and amino acid catabolism-generates abundant immunosuppressive metabolites, including lactate, specific lipid species, and kynurenine. Collectively, these metabolites establish and sustain an immunosuppressive niche through multiple mechanisms: extracellular acidification, driving M2-polarization of TAMs, impairing effector T cell cytotoxicity, and enhancing Treg activity (Figure 2).

### 2.3 Clinical implications of metabolic heterogeneity

The most direct clinical manifestation of metabolic heterogeneity lies in its predictive value for platinum-based chemotherapy sensitivity. This heterogeneity is manifested as a spectrum of glycolytic phenotypes. Tumor cells or regions displaying high aerobic glycolysis-i.e., the Warburg effect, characterized by elevated glucose uptake and lactate production even in the presence of oxygen-exhibit increased glucose uptake and divert pyruvate toward lactate generation. This contrasts with tumor cells with lower glycolytic flux, which may rely more heavily on mitochondrial OXPHOS for energy production. The high glycolytic phenotype not only promotes tumor cell proliferation but also facilitates their evasion of apoptosis (Jin S. et al., 2023). Metabolic imaging (FDG-PET) quantifies tumor glycolytic activity, and radiomic texture features derived from these images offer enhanced characterization of tumor heterogeneity. However, limited spatial resolution may obscure intratumoral heterogeneity (Chicklore et al., 2013; Cook et al., 2018). Integrating FDG-PET with transcriptomic analysis reveals associations between metabolic activity and signaling pathways, enabling prediction of potential therapeutic targets. For instance, transcriptomic profiling of breast cancer (BC) samples stratified by FDG-PET uptake demonstrated strong links between BC metabolism and CXCL8/EGFR signaling activation, driving a shift toward a glycolytic phenotype (Confalonieri et al., 2024). This integrated analytical strategy has demonstrated translational utility. Combining FDG-PET with spatial transcriptomics may thus provide a robust biomarker system to guide precision chemotherapy decisions in ovarian cancer. Therefore, the integration of FDG-PET and spatial transcriptomics holds promise as a powerful biomarker platform for guiding precision chemotherapy strategies in ovarian cancer. This combined analysis enables the stratification of patients into distinct therapeutic subsets. For example, tumors displaying high glycolytic flux (increased FDG uptake) alongside transcriptomic signatures of OXPHOS suppression and upregulated DNA repair pathways may be particularly susceptible to combinations of glycolytic inhibitors (e.g., 2-DG) and PARP inhibitors, leveraging their inherent metabolic vulnerabilities (Sharma et al., 2022; Huang et al., 2025). In contrast, tumors with low FDG-PET avidity and transcriptomic profiles indicative of enhanced FAO and growth factor signaling (e.g., PI3K/AKT pathway) can be targeted combinatorially with FAO inhibitors (e.g., etomoxir) and matched signaling pathway antagonists (Li et al., 2023; Su et al., 2023). Additionally, spatial correlations between metabolic hotspots and immune checkpoint expression can pinpoint regions suitable for combined metabolic-immunotherapeutic strategies, such as the combination of LDHA inhibitors with anti-PD-1/PD-L1 agents (Yang et al., 2025).

Metabolic reprogramming transcends its role as a predictor; it constitutes a fundamental driver of resistance to platinum-based chemotherapy. The metabolic index reflecting glucose-derived lipogenesis and fatty acid uptake directly correlates with cisplatin resistance (Zhang L. et al., 2025). Enhanced fatty acid uptake promotes cisplatin resistance primarily by fueling FAO, which sustains tumor cell survival under cisplatin-induced oxidative stress (Tan et al., 2022). Mimicking *in vivo* metabolic profiles using patient-derived organoids and pharmacologically inhibiting key metabolic enzymes can significantly restore chemosensitivity (Hu and Lazar, 2022; Gilbert et al., 2024). For instance, the use of tumor organoids has led to the development of anti-LGR4 monoclonal antibodies, which effectively induce ferroptosis in chemoresistant organoids when combined with chemotherapy. LGR4 is a G protein-coupled receptor (GPCR) that has been shown to promote fatty acid metabolism and redox homeostasis in cancer cells. Inhibition of LGR4 disrupts these metabolic processes, thereby rendering cells sensitive to chemotherapy-induced ferroptosis (Pei and Luan, 2023). Such metabolism-targeted interventions represent a promising new avenue to overcome chemotherapy resistance in ovarian cancer, accelerating the translation of precision oncology approaches.

Beyond chemotherapy, the heterogeneity of the tumor metabolic microenvironment significantly modulates the efficacy of ICIs (Kao et al., 2022). Tumor regions with high glycolytic flux are characterized by lactate accumulation and hypoxia, leading to extracellular acidosis and functional exhaustion of tumor-infiltrating lymphocytes (TILs) (Calcinotto et al., 2012; Scharping et al., 2021). PD-1 expression on Tregs further enhances their suppressive activity and diminishes immunotherapy efficacy (Kumagai et al., 2022). Within TAMs, hypoxia-induced REDD1 expression suppresses mTOR signaling, consequently impeding glycolysis; this metabolic constraint curtails the pro-angiogenic activity of TAMs, ameliorating dysfunctional vasculature (Wenes et al., 2016). Hypoxia also upregulates PD-L1 on tumor cells and induces other inhibitory receptors on T cells, establishing multifaceted immunosuppressive barriers (Noman et al., 2014; Do et al., 2013). Moreover, CD8^+^ T cells within highly glycolytic niches exhibit impaired mitochondrial OXPHOS, compromising their capacity for sustained anti-tumor activity (Yu et al., 2020). Nutritional interventions aimed at remodeling the immune microenvironment by targeting immune metabolism and metabolic crosstalk within the TME can potentiate anti-tumor responses (Hirschberger et al., 2021; Geiger et al., 2016). Collectively, these mechanisms suggest that combining metabolic modulators with anti-PD-1 therapy offers a viable strategy to counter chemoresistance in ovarian cancer.

**FIGURE 3:**
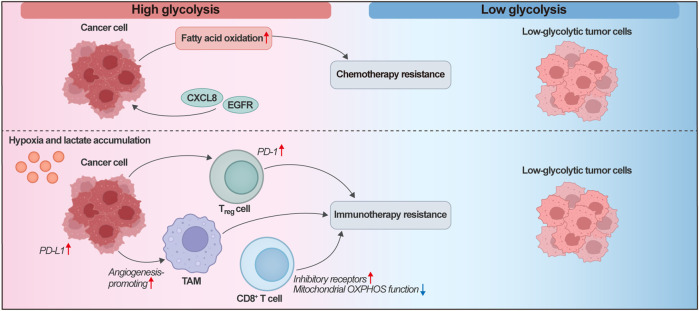
Mechanisms of Metabolic Spatial Heterogeneity Impacting Therapeutic Response in Ovarian Cancer. High-glycolysis cancer cells exhibit hypoxia and lactate accumulation, promoting angiogenesis, PD-L1 expression, and fatty acid oxidation, which contribute to chemotherapy and immunotherapy resistance via CXCR8/EGFR signaling and Treg cell-mediated immunosuppression. Enhanced PD-L1 expression and mitochondrial OXPHOS dysfunction in CD8^+^ T cells further exacerbate immunotherapy resistance. In contrast, tumor cell subsets with low glycolytic activity exhibit weaker therapeutic resistance mechanisms and may represent a more treatment-sensitive component within heterogeneous tumors. The spatial distribution of these metabolic features underscores their role in driving differential treatment outcomes and highlights potential targets for overcoming resistance. PD-L1, Programmed Death-Ligand 1; EGFR, Epidermal Growth Factor Receptor; OXPHOS, Oxidative Phosphorylation; Tregs, Regulatory T cells; TAMs, Tumor-Associated Macrophages.

In conclusion, metabolic heterogeneity in ovarian cancer demonstrates significant clinical value-predicting chemotherapy sensitivity, enabling resistance reversal, and augmenting immunotherapy efficacy (Figure 3). Developing biomarkers based on metabolic features and implementing metabolic modulation strategies will establish a new paradigm for precision therapy in ovarian cancer.

### 2.4 Spatial omics-driven therapeutic strategies targeting subtype-specific metabolic vulnerabilities in ovarian cancer

The major molecular subtypes of ovarian cancer (stromal, immunoreactive, proliferative, and differentiated) exhibit distinct TME characteristics, which determine their unique metabolic dependencies and therapeutic sensitivities. Spatial multi-omics technologies provide an unprecedented perspective for deciphering these subtype-specific metabolic vulnerabilities and guiding precision therapy.

The stromal subtype is characterized by a dense stroma enriched with CAFs. Spatial metabolomic analysis may reveal that CAFs produce lactate via glycolysis, while adjacent tumor cells potentially utilize this lactate for OXPHOS, forming a putative “metabolic symbiosis.” This finding highlights the therapeutic value of targeting lactate transporters (MCT1/MCT4) (De la Cruz-López et al., 2019; Gupta et al., 2017). Additionally, tumor spheroids of this subtype may rely on macropinocytosis to acquire nutrients in the nutrient-depleted ascites microenvironment, offering an exploratory direction for targeting this pathway.

Although the immunoreactive subtype is marked by abundant immune cell infiltration, these cells are often functionally suppressed. Spatial transcriptomic analysis may uncover that tumor cells and MDSCs in this subtype highly express arginase 1 (ARG1) and IDO1, which may deplete arginine and tryptophan in the TME, leading to T cell dysfunction (Mondanelli et al., 2019). This provides a novel rationale for combining ICIs with metabolic enzyme inhibitors. Meanwhile, the glycolytic activity of tumor cells may generate a lactic acid microenvironment that further impairs T cell function, suggesting that strategies such as MCT inhibitors warrant further investigation (Lopez et al., 2023).

The proliferative subtype is defined by high tumor cell proliferative activity, which may increase the demand for biosynthetic precursors such as nucleic acids, lipids, and amino acids (Martínez-Reyes and Chandel, 2021). Spatial metabolic imaging may demonstrate active serine-glycine metabolic pathways and nucleotide synthesis in this subtype. This metabolic signature indicates potential sensitivity to targeted anabolic interventions, such as PHGDH inhibitors (serine synthesis) and DHODH inhibitors (pyrimidine synthesis).

In summary, spatial multi-omics studies hold great promise for unraveling the metabolic features of different ovarian cancer subtypes and may guide the development of subtype-specific therapeutic strategies, providing new research directions to overcome the limitations of current treatments.

## 3 Spatial profiling of the ovarian cancer immune microenvironment: from cellular architecture to functional networks

### 3.1 Spatial architecture of immune cell distribution

Effective regulation of the ovarian cancer immune microenvironment hinges on the precise spatial patterning of immune cells. Cutting-edge spatial profiling technologies-including GeoMx® Digital Spatial Profiler (DSP) and CODEX®-enable comprehensive mapping of immune cell subsets (e.g., T/B lymphocytes, macrophages, CAFs) within distinct anatomical niches: the tumor core, invasive margin, and tertiary lymphoid structures (TLSs). These platforms quantify cellular localization, abundance, and interaction networks, providing a critical topological framework for deciphering heterogeneity in immune responses and mechanisms of therapeutic resistance.

TLSs function as pivotal hubs for anti-tumor immunity, where their spatial distribution and maturation status profoundly impact patient prognosis. In ovarian cancer, TLS formation involves chemokine-mediated lymphocyte recruitment (e.g., CXCL13, CCL19/21) and localized microenvironmental remodeling (Sautès-Fridman et al., 2016). Within TLS germinal centers (GCs), B cell differentiation, antibody secretion, and T cell activation are supported, amplifying anti-tumor immunity (Victora and Nussenzweig, 2022). TLSs foster tumor antigen-specific responses while limiting immunosuppressive cell infiltration, correlating significantly with improved patient survival (Munoz-Erazo et al., 2020; Colbeck et al., 2017; Dieu-Nosjean et al., 2014). Spatial multi-omic analysis of cholangiocarcinoma via GeoMx DSP revealed concentrated clusters of CD19^+^ B cells and CD3^+^ T cells within TLSs. Furthermore, the top differentially upregulated genes between TLSs and TILs were predominantly B cell-associated, suggesting synergistic anti-tumor activity mediated through antigen presentation, co-stimulatory signaling, and cytokine exchange (Chung et al., 2025). GeoMx DSP-based multiplexed proteomic profiling of platinum-refractory (PRF) and platinum-sensitive (PS) advanced high-grade serous ovarian cancer identified elevated apoptosis and anti-tumor immune signatures in PS patients, contrasting with PRF tumors exhibiting AKT1 and WNT dual signaling indicative of immunosuppression (Scalise et al., 2024). Quantifying these spatial interaction networks not only offers novel perspectives for developing TLS-based biomarkers to guide personalized immunotherapies in ovarian cancer but also deepens our understanding of tumor-immune crosstalk within the TME.

Moving beyond the localized effects of TLSs, the immune landscape across distinct functional tumor regions-such as the core versus invasive margin-exhibits profound heterogeneity, sculpting unique immunosuppressive or immunopermissive niches. Significant spatial divergence characterizes the immune microenvironments of the tumor core and invasive front (Wu et al., 2023a). Within the invasive niche, malignant cell-induced stressed stromal cells secrete serum amyloid A (SAA) proteins, recruiting M2-polarized macrophages. A functional triad emerges: highly invasive CXCL6^+^ tumor cells, SAA^+^ stromal cells, and recruited FPR1^+^ M2 macrophages interact synergistically to drive tumor progression (Wu et al., 2023a). Macrophages and NK/T cells accumulate at the tumor-stroma interface, while fibroblasts exhibit pronounced enrichment. This spatial patterning potentially links to hypoxia-driven metabolic reprogramming and HIF-1α-mediated upregulation of immune checkpoint molecules, collectively constraining anti-tumor immunity (Wu et al., 2023a; Ni et al., 2020). CODEX™-based spatial multi-omics imaging delineates immunologically active (“hot”) versus excluded (“cold”) peritumoral niches surrounding spatial subclones and identifies novel immune exhaustion markers (Mo et al., 2024). Concurrently, CODEX integrated with spatial proteomics reveals remarkable plasticity within cancer-CAF subpopulations (Foster et al., 2022). These findings elucidate spatial tumor evolution through local microenvironmental interactions, uncovering patterns of spatial co-localization between M2 macrophages and CAFs within fibrotic regions. This provides a novel biological rationale for targeting immunosuppressive hubs in ovarian cancer.

The ultimate clinical value of immune cell spatial architecture lies in its predictive power for immunotherapy response. This underpins microenvironmental classifications-immune-desert, -inflamed, and -excluded phenotypes-which form the bedrock for precision immunotherapy (Gerard et al., 2021). Immune-desert regions lack significant immune infiltration and correlate with therapy resistance. Inflamed zones display abundant yet functionally constrained immune cells. The excluded phenotype manifests as immune cells confined to stromal cuffs, physically segregated from tumor cells (Hegde et al., 2016; Tumeh et al., 2014; Gajewski et al., 2013). Integrating pan-tissue spatial transcriptomics with single-cell multi-omics identified four conserved CAF spatial subtypes. Notably, s1-CAFs-highly expressing ACTA2 and TGFB1-mediate matrix remodeling and immunosuppression, potentially generating excluded or desert microenvironments that diminish immune checkpoint inhibitor efficacy (Liu et al., 2025). This positions microenvironmental subtyping as a predictive biomarker strategy to guide personalized ovarian cancer therapy.

Furthermore, the spatial architecture of the ovarian cancer immune microenvironment extends beyond solid tumor masses to encompass the critical compartment of malignant ascites. Ascites serves as a dynamic “liquid TME” that facilitates peritoneal dissemination. Although the cellular heterogeneity and lack of inherent spatial structure of malignant ascites present significant research challenges, emerging spatial multi-omics technologies are providing groundbreaking solutions. First, by integrating single-cell data from ascites with spatial transcriptomic and proteomic data from primary tumors and omental tissues, we can trace the “origin” of tumor cells in ascites and identify shed cell clones with high metastatic potential along with their key survival genes. Second, spatial transcriptomic or multiplex immunofluorescence analysis of pelleted ascites cell blocks enables precise dissection of the internal architecture of tumor spheroids, revealing metabolic gradients such as the “hypoxic-necrotic core and proliferative outer shell,” while mapping the scaffold roles of CAFs and mesothelial cells, as well as the spatial distribution of immune cells-for example, whether T cells are excluded, trapped, or entirely absent. Finally, using Laser Capture Microdissection (LCM) or high-resolution DSP, specific micro-regions-from the mesothelial layer and newly attached spheroids to established metastatic lesions on the peritoneum-can be precisely isolated and molecularly decoded. This approach dynamically captures the “cellular dialogue” driving tumor attachment and systematically elucidates the central role of ascites in remodeling the metastatic process in ovarian cancer.

**FIGURE 4:**
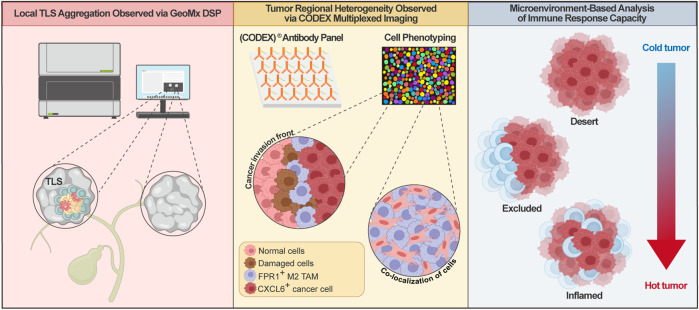
Spatial Topological Structure of the Ovarian Cancer Immune Microenvironment. The left panel shows local tertiary lymphoid structure (TLS) aggregation observed via GeoMx DSP, indicating regions of enhanced immune activity. The middle panel, captured with CODEX multiplexed imaging and antibody panel, depicts tumor regional heterogeneity, including the invasion front with normal cells, cancer-associated cells, FPR1+ M2 TAMs, and CXCL6+ cancer cells. The right panel presents microenvironment-based analysis of immune response capacity, classifying tumor regions into inflamed (hot tumor), excluded, and desert (cold tumor) phenotypes based on immune infiltration and responsiveness, providing insights for spatially guided immunotherapeutic strategies. TLS, Tertiary Lymphoid Structure; TAMs, Tumor-Associated Macrophages.

In summary, spatial orchestration of immune cells in ovarian cancer-modulated through TLS activation, regional heterogeneity, ascites-mediated dissemination, and microenvironmental classification-dictates immune responsiveness (Figure 4). Deciphering these spatial features provides a critical theoretical foundation for developing immune-topography-guided therapeutic strategies and prognostic biomarkers.

### 3.2 Spatiotemporal regulation of immunometabolic checkpoints

The expression and activity of immune checkpoint molecules (e.g., PD-L1, adenosine pathway components) are not static but are dynamically fine-tuned by the local tumor metabolic microenvironment, giving rise to spatially compartmentalized “immunometabolic checkpoints.” Deconstructing the spatiotemporal regulatory networks through which metabolic signals govern immune checkpoints represents a critical nexus for understanding the spatial heterogeneity of immune evasion in ovarian cancer, overcoming immunotherapy resistance, and devising novel combinatorial strategies.

As the most intensively studied immune checkpoint, PD-L1 expression is subject to intricate regulation by diverse metabolic pathways, with hypoxia-driven glycolysis and lactate metabolism playing pivotal roles. Research demonstrates a strong link between PD-L1 expression on tumor cells and metabolic reprogramming. Specifically, HIF-1α drives aberrant PD-L1 upregulation by activating glycolytic pathways, establishing a synergistic network coupling metabolic signaling to immune evasion. In a GL261 syngeneic glioma model in wild-type C57BL/6 mice, HIF-1α inhibition (PX-478, 30 mg/kg daily) combined with anti-PD-L1 antibody (10 mg/kg twice weekly) synergistically reduced tumor volume by >60%, enhanced CD8^+^ T cell activation (IFN-γ^+^), and improved survival versus monotherapy (n = 5/group, P < 0.01) (Ding et al., 2021). Lactate indirectly sustains stable PD-L1 expression through mechanisms including microenvironmental acidification and activation of G protein-coupled receptors (e.g., GPR81) (Feng et al., 2017). Critically, inhibiting the glycolytic enzyme LDHA or blocking the lactate transporter MCT4 attenuates PD-L1-mediated immunosuppression, thereby bolstering the clinical feasibility of enhancing anti-tumor immune responses (Cui et al., 2025; Babl et al., 2023). This bidirectional immunometabolic crosstalk not only explains primary resistance to ICIs in highly glycolytic tumors but also provides a mechanistic rationale for combination therapies targeting the HIF-1α/PD-L1 axis or lactate metabolism.

Beyond the PD-1/PD-L1 axis, the adenosine signaling pathway represents another crucial spatially defined immunometabolic checkpoint, centered on the spatial remodeling of the extracellular ATP-adenosine metabolic gradient. The adenosine pathway (CD39/CD73/ADO) exerts potent immunosuppression within the TME by regulating extracellular ATP catabolism to adenosine. CD39 and CD73 sequentially catalyze ATP degradation to adenosine, which subsequently triggers immunosuppression via A2a receptor activation (Vijayan et al., 2017; Klysz et al., 2024). Spatial metabolomics provides precise quantification of the ATP-adenosine metabolic gradient within ovarian tumor tissues. This mapping reveals specific immunosuppressive phenotypes spatially correlated with adenosine-enriched niches, offering topographical guidance for targeted interventions. This mapping provides critical topographical guidance for targeted interventions. Small molecule inhibitors or antibodies targeting CD73 can block adenosine generation, reversing the immunosuppressive milieu (Young et al., 2016). Combining these agents with PARP inhibitors-which enhance tumor antigen release by inducing DNA damage-synergistically promotes effector T cell infiltration and functional restoration (Besse et al., 2024). Preclinically, the CD73 inhibitor CB-708 demonstrates enhanced anti-tumor activity when combined with anti-PD-L1 antibodies (Lee et al., 2019), suggesting this combinatorial strategy holds promise for overcoming resistance to existing ICIs in ovarian cancer.

Advances in spatial profiling technologies are driving deeper understanding of the metabolic regulation and spatial heterogeneity of immune checkpoints, offering unprecedented insights into tumor evolution and precision targeting. During tumorigenesis, the levels and spatial distribution of markers for dedifferentiation, proliferation, and immune checkpoints undergo significant remodeling, reflecting dynamic TME evolution and its impact on disease progression (Sheng et al., 2022). Notably, metabolic polarization of immune cells toward the tumor-immune boundary occurs in some tissues, accompanied by increased expression of transporters like CD98 and ASCT2, which influence tumor aggressiveness and therapy resistance (Hartmann et al., 2021; Shimizu et al., 2014; Kaira et al., 2009). Spatial omics technologies, by integrating spatially resolved gene expression, protein localization, and metabolic signatures, provide a comprehensive framework for dissecting ovarian cancer TME heterogeneity, yielding novel perspectives on tumor initiation, progression, and metastasis.

Collectively, elucidating the spatiotemporal metabolic regulation of PD-L1 and the adenosine pathway, particularly through the lens of spatial omics, not only provides a multidimensional theoretical foundation for understanding immune evasion but also paves the way for precision combination strategies that co-target these immunometabolic checkpoints to overcome therapeutic resistance in ovarian cancer.

### 3.3 Stromal regulation of the metabolic-immune axis

Stromal elements within the ovarian cancer microenvironment-including CAFs, adipocytes, and endothelial cells-transcend structural support to function as pivotal regulators of the metabolic-immune axis. Through secretion of specific metabolites and cytokines, alongside physical barrier formation, they spatiotemporally remodel local metabolic niches, directly and indirectly modulating immune cell functionality and distribution. This orchestration profoundly influences disease progression and therapeutic resistance.

As the most metabolically active stromal component, CAFs architect immunosuppressive metabolic landscapes primarily via secretion of key metabolites like lactate and glutamine. CAFs remodel the TME through lactate and glutamine efflux, establishing metabolic niches conducive to tumor advancement (Pavlides et al., 2009; Francescone et al., 2021). Lactate operates as both an energetic substrate and signaling molecule: its acidification of the microenvironment enhances tumor glycolytic flux and immune evasion, as shown in Pan02 pancreatic xenografts in C57BL/6 mice and NSCLC xenografts tracing 13C-lactate uptake (Martinez-Outschoorn et al., 2017; Chen et al., 2025; Certo et al., 2021), while glutamine provision furnishes carbon/nitrogen sources for tumor cells, fueling nucleotide biosynthesis and redox homeostasis (Altman et al., 2016). Spatial multi-omics profiling of pancreatic cancer following neoadjuvant chemotherapy (NAC) revealed elevated expression of metabolites (e.g., proline, glycine) within the immune-TME interface. Concurrently, significant metabolic reprogramming occurred-downregulation of key glycolytic enzymes alongside upregulation of lipid metabolism, glutaminolysis, and OXPHOS components (Tang et al., 2023). These findings not only elucidate linkages between NAC-induced metabolic reconfiguration and immune microenvironment alterations but also provide a mechanistic foundation for developing precision strategies co-targeting metabolic pathways (e.g., CD36-mediated lipid scavenging) and immunotherapy.

Beyond CAFs, adipocytes-leveraging their distinctive lipid metabolic signature-emerge as significant contributors to immunosuppression within specialized niches like ovarian cancer omental metastases. Recent studies reveal that tumor-associated adipocytes release unsaturated fatty acids that activate the peroxisome proliferator-activated receptor gamma (PPARγ) pathway, driving macrophage polarization toward an anti-inflammatory M2 phenotype. Concurrently, they suppress NF-κB signaling, attenuating pro-inflammatory cytokine secretion, thereby orchestrating localized immunosuppression (Wang et al., 2025; Scirpo et al., 2015). This immunometabolic interplay is particularly pronounced in malignancies prone to omental dissemination, including ovarian cancer (Wang et al., 2024; Tan et al., 2018). MALDI analysis of human omental tumor sections demonstrated heightened phosphatidylcholine abundance in cancer cells proximal to adipocytes, contrasting with distal cells enriched in phosphatidylinositol (PI). This spatially resolved metabolomics confirms adipocyte-mediated phosphatidylcholine elevation in neighboring tumor cells (Mukherjee et al., 2023). Collectively, these findings provide experimental justification for targeting adipocyte metabolism in ovarian cancer immunotherapy strategies.

Endothelial cells spatially navigate immune cell trafficking and positioning through their metabolic state and chemokine secretion, profoundly sculpting regional immune responses. Endothelial metabolism critically regulates vascular functionality and directs lymphocyte delivery/activation (Scharping et al., 2021; Li et al., 2018; Haas et al., 2015; Tyrakis et al., 2016; Riesenberg et al., 2007). In 3D models co-culturing human atherosclerotic plaques with autologous CD8^+^ T cells, T cells preferentially localized near neovessels. CXCL12-overexpressing endothelia within these vessels governed CD8^+^ T cell migration (Parma et al., 2025). Integrated single-cell and spatial transcriptomics in oral squamous cell carcinoma revealed Treg enrichment within high-metabolism CAF domains (Liu Z. et al., 2024). Notably, inflammatory CAFs (iCAFs)-exhibiting elevated pyruvate carboxylase expression-recruit Tregs via CXCL12 secretion, establishing immunosuppressive hubs (Ma et al., 2023; Schwörer et al., 2021). Therefore, endothelial metabolic reprogramming constitutes a promising approach to regulate immune cell infiltration and activation, laying a theoretical foundation for developing novel combined anti-angiogenic/immunotherapeutic strategies in ovarian cancer.

Through metabolite secretion and spatial signaling, CAFs, adipocytes, and endothelial cells collaboratively construct a stromal network governing the metabolic-immune axis. The inherent spatial heterogeneity of this network unveils novel therapeutic targets for stroma-directed immunotherapies.

## 4 Spatial multi-omics technologies: novel tools for deconvoluting the metabolic-immune axis

The selection of multi-omics technologies discussed in this review-particularly spatial transcriptomics, proteomics, and metabolomics-is based on a core hypothesis: the functional output of the metabolism-immune axis is not merely the sum of its parts, but arises from spatial synergy at the molecular level. While genomics provides a blueprint for potential metabolic perturbations (e.g., mutations in metabolic enzymes), it fails to capture the dynamic nature, post-translational modifications, and microenvironment-dependent characteristics of immuno-metabolic crosstalk. We therefore prioritize technologies that address this gap:

Spatial transcriptomics (e.g., Xenium, Visium): Critical for localizing the expression of metabolic enzymes (e.g., HK2, LDHA), immune checkpoint ligands (e.g., PD-L1), and chemokines within the structural context of the TME, revealing ‘who is where and what they might be doing.'

Spatial proteomics (e.g., GeoMx DSP, CODEX): Since the localization and abundance of proteins-especially metabolic transporters (e.g., MCTs, CD98) and immune cell surface markers (e.g., CD3, CD68)-are often not directly correlated with mRNA levels, spatial proteomics enables direct validation of pathway activity and intercellular interactions.

Spatial metabolomics (e.g., MALDI-MSI, DESI): Represents the ultimate functional readout, providing direct evidence of the biochemical consequences of metabolic reprogramming (e.g., lactate and kynurenine gradients) that directly suppress immune cell function. This level of information cannot be inferred solely from transcriptomic or proteomic data.

These specific technologies were therefore selected to decode functional spatial networks, from gene expression to protein function, and ultimately to metabolite-mediated cellular crosstalk.

### 4.1 Breakthroughs in spatial metabolomics

The rapid evolution of spatial metabolomics technologies-particularly breakthroughs in high-resolution imaging and *in situ* metabolite detection-provides revolutionary tools for unbiased, spatially resolved mapping of metabolite distributions within the native tissue microenvironment of ovarian cancer. These advances enable direct visualization of metabolic heterogeneity, dynamic tracking of metabolic flux, and the establishment of spatial correlations between metabolic landscapes, immune phenotypes, and therapeutic responses. Consequently, they offer unprecedented capacity for definitive elucidation of metabolic-immune axis crosstalk networks (Table 1).

**TABLE 1:** Comparison of spatial multi-omics technologies.

Technology	Principle	Application	Resolution	Throughput	Analysis method	Clinical applications	Pros & cons
MALDI-MSI	Matrix-assisted laser desorption/ionization	Spatial metabolomics (small molecules, metabolites, lipids, proteins, etc.)	10–100 μm	Moderate (suitable for small-scale sample analysis)	Mass spectrometry-based data analysis; requires specialized software for molecular identification and image reconstruction	Tissue metabolomics, drug distribution studies, cancer biomarker discovery	Pros: Label-free, compatible with diverse molecular types Cons: Limited spatial resolution, complex sample preparation
DESI-MS	Electrospray ionization	Spatial metabolomics (small molecules, metabolites, lipids, etc.)	50–200 μm	Low (suitable for rapid *in situ* detection)	Mass spectrometry-based data analysis; requires specialized software for molecular identification and image reconstruction	*In situ* metabolite analysis, drug metabolism studies, forensic science	Pros: No sample pretreatment required, ideal for *in situ* analysis. Cons: Lower resolution and sensitivity
GeoMx DSP	Probe-based *in situ* hybridization Antigen-antibody interaction	Spatial transcriptomics Spatial proteomics	1–10 μm	High (supports large-scale samples and high-dimensional analysis)	Fluorescence signal deconvolution and multidimensional data analysis; requires advanced bioinformatics tools	Tumor microenvironment analysis, immunohistochemistry, spatial transcriptomics	Pros: High resolution, multiplex target analysis Cons: Probe-dependent, high cost
CODEX	Multiplexed fluorescent antibody labeling Cyclic imaging and deconvolution	Spatial proteomics	1–10 μm	High (supports multiplex protein labeling and cyclic imaging)	Fluorescence signal deconvolution and multidimensional data analysis; requires advanced bioinformatics tools	Immune microenvironment studies, cell-cell interaction analysis, high-dimensional proteomics	Pros: High-dimensional proteomics at single-cell resolution Cons: Complex workflow, demanding data analysis
Xenium	*In situ* RNA sequencing High-throughput fluorescent probes and imaging	Spatial transcriptomics	0.2 μm	High (supports high-throughput single-cell RNA analysis)	*In situ* sequencing data analysis; requires high-throughput data processing and single-cell analysis tools	Single-cell spatial transcriptomics, developmental biology, neuroscience	Pros: Single-cell resolution, high-throughput RNA analysis Cons: Expensive, complex data analysis

Matrix-assisted laser desorption/ionization mass spectrometry imaging (MALDI-MSI), a cornerstone spatial metabolomics platform, has achieved transformative breakthroughs in circumventing clinical sample limitations and visualizing therapy-resistant metabolite topography. Its application to ovarian cancer provides an indispensable tool for spatially resolving chemotherapy resistance-associated metabolomes. MALDI-MSI enables high-resolution mapping and analysis of lipid distributions directly within tumor sections, revealing dynamic lipid metabolic rewiring during acquired resistance (Berry et al., 2011; Fernández et al., 2011). Studies confirm that ovarian cancer chemoresistance correlates strongly with lipid reprogramming; for instance, lysophosphatidic acid (LPA) functions as a negative regulator of type I interferon (IFN) signaling to confer resistance (Chae et al., 2022). Crucially, MALDI-MSI visually contrasts lipid metabolic heterogeneity between resistant and sensitive tumors, identifying resistance-specific lipid biomarkers (Barré et al., 2018). Furthermore, integrated metabolomic reconstruction of FFPE specimens with MALDI-MSI overcomes traditional fresh-tissue dependency, preserving and reactivating lipid metabolic data-thereby vastly expanding clinically relevant sample resources for ovarian cancer research (Ly et al., 2016). This combined approach furnishes critical technical support for delineating ovarian cancer’s metabolic-immune-therapeutic resistance network.

In contrast to MALDI-MSI, desorption electrospray ionization mass spectrometry (DESI-MS) leverages its matrix-free operation to offer unique advantages for high-resolution molecular spatial analysis of live or intraoperative specimens, particularly in elucidating dynamic lipid-immune phenotype correlations. DESI-MS excels at decoding spatiotemporal relationships between lipid metabolic heterogeneity and immune landscapes within the TME (Razo et al., 2024), enabling correlation of lipid distribution patterns with histopathologically defined tumor regions (Awad et al., 2024). Multi-omics analyses implicate dysregulated phospholipid pathways (e.g., phosphatidylcholine and sphingolipid metabolism) in immunosuppressive niche formation (Ping et al., 2024; Soula et al., 2024), suggesting metabolic reprogramming modulates immune effector functions to drive progression. Integrated metabolomic-lipidomic profiling reveals stark differences in tumor transcriptomes, immune infiltrates, fecal microbiomes, and metabolomes between bevacizumab responders and non-responders (Rosario et al., 2024). These insights pioneer metabolism-based stratification frameworks, simultaneously advancing fundamental understanding of immunometabolic crosstalk and laying a translational foundation for optimizing bevacizumab regimens through rational combination therapies.

Beyond static distribution mapping, the integration of metabolic flux tracing with spatial imaging represents a frontier breakthrough in spatial metabolomics, enabling high spatiotemporal resolution analysis of *in vivo* metabolic flux dynamics. The synergy between metabolic flux analysis (e.g., ^13^C-glucose tracing) and spatial imaging platforms provides unprecedented tools for dynamically resolving metabolic networks with high spatial and temporal precision. The ^13^C-SpaceM methodology leverages ^13^C-labeled glucose to trace glucose-dependent *de novo* lipogenesis pathways, achieving single-cell mass spectrometry imaging via ultrahigh-resolution MALDI. By emulating saponification reactions through all-ion fragmentation (AIF) mode, it enables comprehensive co-analysis of fatty acid moieties within major phospholipids including glycerophospholipids (Tang et al., 2023). Ultra-sensitive high-resolution mass detectors deliver precise qualitative and quantitative assessment of fatty acid isotopologues at single-cell resolution (Buglakova et al., 2024). Concurrently, spatial imaging techniques (mass spectrometry imaging, stimulated Raman scattering, or single-cell metabolomics) integrate spatial metabolite distributions to reveal flux heterogeneity across tissue microenvironments and subcellular compartments (Maimó-Barceló et al., 2025; Zhang et al., 2019; Nunes et al., 2024). This multidimensional spatiotemporal profiling empowers researchers to dynamically visualize molecular details of metabolic reprogramming during ovarian carcinogenesis and cellular differentiation-including complex phenomena like metabolic competition within the TME and immunometabolic switching in immune cells.

MALDI-MSI, DESI-MS, and integrated metabolic flux-spatial imaging platforms synergistically deliver multidimensional analytical frameworks for ovarian cancer research-deciphering metabolite topography, lipid metabolic networks, and spatiotemporal dynamics to resolve spatial heterogeneity within the metabolic-immune axis.

### 4.2 Advances in spatial immunomics

Rapid advancements in spatial immunomics now enable precision mapping and quantitative analysis of immune cell composition, functional states, and interaction networks directly within intact tissue architecture. These technologies overcome the spatial information deficit inherent to conventional single-cell sequencing, providing unprecedented capacity to resolve the complex spatial organization, heterogeneity, and dynamic interplay with metabolic reprogramming and therapeutic responses within the ovarian cancer immune microenvironment.

Within spatial proteomics, the GeoMx® DSP has emerged as a pivotal platform for decoding immune checkpoint spatial heterogeneity through its region-specific, high-plex analytical capabilities. As a next-generation spatial proteomic technology, GeoMx DSP facilitates high-resolution, multiplexed protein expression analysis within morphologically defined tissue regions. This innovative approach precisely localizes distinct functional compartments-including tumor core, immune infiltrates, and stromal zones-via integrated laser microdissection and fluorescent barcoding, while quantifying spatial disparities in key immune checkpoint protein expression (Merritt et al., 2020; Hernandez et al., 2022). When coupled with high-plex proteomics (40+ targets), it achieves multiplexed quantification of >40 antigens at subcellular resolution, constructing multidimensional immune regulatory network maps (Wu et al., 2022). This spatially resolved proteomic strategy delivers critical scientific rationale for optimizing immunotherapy response prediction and developing combination therapies targeting immunosuppressive niches, thereby propelling translational research in precision immuno-oncology for ovarian cancer.

Imaging mass cytometry (e.g., CODEX) and super-resolution spatial transcriptomics (e.g., Xenium) deliver spatially resolved protein or gene expression profiles at single-cell resolution, enabling precise mapping of cellular interactions and functional states. CODEX generates spatially resolved single-cell protein maps (Goltsev et al., 2018), delineating the immune tumor microenvironment in lung cancer and revealing cellular/molecular progression from precancerous lesions to malignancy (Rozenblatt-Rosen et al., 2020). Analysis of tumors from 25 bladder cancer patients via CODEX identified PD-L1/PD-L2-expressing CDH12^+^ epithelial cells co-localizing with CD8^+^ T cells-a spatial configuration potentially underlying clinical responses to checkpoint inhibitors (Gouin et al., 2021). Xenium-based spatial transcriptomics of small intestine tissue mapped tissue-resident memory (TRM) cell distributions across villus tips and crypt bases, uncovering significant transcriptional differences between these niches (Reina-Campos et al., 2025). This study pioneers a paradigm that combines spatial multi-omics with metabolic pathway analysis, uncovering how distinct tissue microenvironments (e.g., intestinal villi and crypts) modulate the metabolic phenotype and functional properties of TRM cells. This serves as a critical point of reference for deciphering T cell adaptation within the TME. While the research focuses on the healthy gut, the methodologies it showcases and the concept of “metabolic checkpoints”-the regulation of T cell function by tissue-specific metabolic niches-provide a conceptual framework and research paradigm for investigating analogous metabolism-centered combination immunotherapeutic approaches in ovarian cancer.

Beyond transcriptional/proteomic profiling, spatial epigenomics (e.g., ATAC-seq) resolves spatial heterogeneity in chromatin accessibility, offering novel insights into epigenetic regulation of immune cell functional states. Spatial ATAC-seq interrogates genome-wide chromatin accessibility landscapes (Buenrostro et al., 2013), mapping regulatory element dynamics to elucidate epigenetic mechanisms in development and pathology. Applied spatially, it discriminates compartment-specific epigenetic signatures in lymphoid organs (Li et al., 2022) and identifies pathogenic chromatin architectures in autoimmunity (e.g., hyperaccessible inflammatory loci in lupus) (Perez et al., 2022; Yu et al., 2021). Comparative analysis of tumor-infiltrating versus peripheral lymphocytes further reveals epigenetic dysregulation during T cell exhaustion following immunotherapy (Sundaram et al., 2024; Satpathy et al., 2019). This methodology holds promise for identifying epigenetic regulators of immune checkpoints to guide targeted therapies for ovarian cancer.

Collectively, GeoMx DSP, CODEX, and spatial ATAC-seq provide multidimensional frameworks for investigating spatial heterogeneity along the metabolism-immunity axis in ovarian cancer through protein mapping, single-cell.

### 4.3 Integrative multi-omics frameworks

While single-modality spatial omics provide localized insights, deciphering ovarian cancer’s metabolism-immunity network necessitates systematic integration of multimodal spatial data. Emerging computational frameworks are advancing spatial multi-omics beyond mere data superposition toward true information fusion, enabling system-level analysis spanning molecular features, spatial coordinates, and functional interactions. This paradigm shift offers unprecedented opportunities for constructing precision disease models and guiding clinical decision-making.

Next-generation AI algorithms (e.g., SpatialGlue) are redefining integration paradigms through adaptive cross-modal coupling. Unlike traditional methods reliant on preprocessing-stage stitching, SpatialGlue establishes a spatially guided multimodal framework via bidirectional attention mechanisms. This architecture dynamically resolves feature-coordinate interdependencies within a unified computational space-autonomously quantifying modality-specific contributions (e.g., transcriptome vs. proteome) while precisely capturing microenvironmental couplings (e.g., cellular neighborhood topology with gene expression gradients). Such hierarchical attention overcomes fixed-weight fusion limitations, enabling biologically adaptive integration (Long et al., 2024). Applied to ovarian cancer multi-omics (metabolome, transcriptome, proteome), this approach can reconstruct metabolism-immunity interaction networks to systematically resolve dynamic crosstalk between metabolic reprogramming and immune regulation. Deep learning models leveraging these multidimensional features, when integrated with histopathological and spatial molecular data (Chen et al., 2022; Gao et al., 2025), may predict therapy-sensitive niches via graph neural networks and attention mechanisms. This quantifies treatment response heterogeneity across cellular subpopulations while providing an extensible analytical framework for solid tumors.

Integrated multi-omics analyses (genomics, transcriptomics, proteomics, metabolomics, spatial omics) demonstrate significant potential for disease subtyping and clinical translation, offering innovative frameworks for precision oncology in malignancies like ovarian cancer. By consolidating genomic, transcriptomic, proteomic, and metabolomic profiles with spatial transcriptomic/metabolomic data and immune microenvironment features, recent work classified renal cell carcinoma into four distinct immune subtypes-providing actionable strategies for precision therapy (Hu et al., 2024). Clinically, spatial multi-omics elucidates spatial correlations between metabolic heterogeneity and immune infiltration in ovarian tumors (Schweizer et al., 2023; Kader et al., 2024), informing immunophenotyping while enabling precise delineation of target volumes where hypermetabolic zones overlap with immunosuppressive microenvironments. This facilitates optimized radiation dosing during local therapy planning while sparing normal tissues. Complementarily, live-organoid spatiotemporal (LOST) technology employs 3D organoid models coupled with spatial transcriptomics to simulate dynamic tumor microenvironment responses, revealing critical cell-cell communication networks (Ma S. et al., 2024). These advances hold substantial implications for organoid-based precision medicine and drug development.

Despite promise, spatial multi-omics integration faces significant technical and translational hurdles requiring coordinated advances. Current resolution remains constrained by detection sensitivity and physical limits (Si et al., 2025), while dynamic process capture is hampered by destructive sample processing, preventing reconstruction of continuous spatiotemporal processes like cell migration (Liu et al., 2020). Clinically, heterogeneous analytical pipelines impede cross-institutional data integration, and most findings remain mechanistic-lacking validated pathways from spatial biomarker discovery to diagnostic applications (Liu et al., 2021; Vandereyken et al., 2023). Future efforts will fuse next-generation imaging that approaches sub-organelle resolution with single-cell multi-omics, generating three-dimensional molecular maps at the nanoscale. Concomitantly, we will devise *in-situ* live-cell labelling strategies and temporal modelling algorithms, integrating organoid systems to create a four-dimensional spatial-temporal atlas. These advances will feed directly into an automated spatial-pathology platform, a pan-cancer repository of spatial heterogeneity, and prospective trials that rigorously test the prognostic power of spatial biomarkers.

## 5 Precision targeting of the metabolic-immune crosstalk

### 5.1 Combinatorial metabolic and immune modulation

**FIGURE 5:**
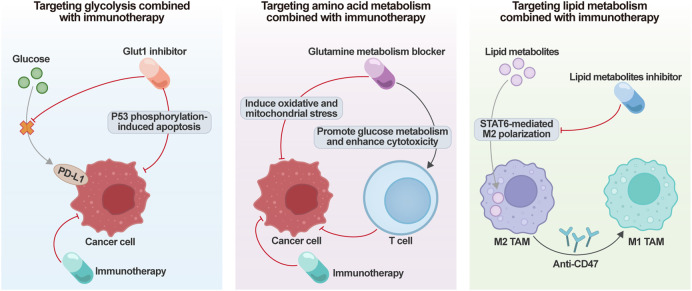
Combined Therapeutic Strategies Targeting the Metabolism-Immune Axis. The left panel presents targeting glycolysis combined with immunotherapy: A glycolysis inhibitor disrupts tumor cell glycolytic metabolism, which not only blocks glucose-dependent PD-L1 upregulation but also directly triggers tumor cell apoptosis through p53 phosphorylation. Concurrently, immunotherapy overcomes residual PD-L1-mediated immune evasion. The middle panel depicts targeting amino acid metabolism (glutamine) combined with immunotherapy: A glutamine metabolism inhibitor induces oxidative stress and mitochondrial dysfunction in tumor cells; this, in turn, feedback-enhances T cell glucose metabolic activation and cytotoxicity, enabling reactivated T cells to effectively attack and eliminate metabolically impaired tumor cells. The right panel shows targeting lipid metabolism combined with immunotherapy: a lipid metabolites inhibitor suppresses STAT6-mediated M2 polarization signaling in tumor-associated macrophages (TAMs), and an anti-CD47 antibody remodels macrophage function to reprogram immunosuppressive M2 TAMs into anti-tumor M1 phenotypes, cooperating with immunotherapy to eliminate tumor cells. PD-L1, Programmed Death-Ligand 1; TAMs, Tumor-Associated Macrophages.

The metabolically driven immunosuppressive landscape in ovarian cancer constitutes a fundamental resistance mechanism to immunotherapies. Strategic targeting of core metabolic circuits-glycolysis, glutamine metabolism, and lipid pathways-to reprogram the immune microenvironment, coupled with immune checkpoint blockade, has emerged as a precision approach to enhance treatment efficacy (Figure 5).

Coordinated glycolysis inhibition and checkpoint disruption exhibits compelling therapeutic synergy. Glut1-targeting agents combined with PD-1 inhibitors significantly restrict tumor progression in preclinical models, as demonstrated in C57BL/6 mice bearing LLC or B16F10 tumors, where BAY-876 plus anti-PD-1 treatment robustly suppressed tumor growth in an immune-dependent manner (Wu et al., 2023b). Cancer cells exploit glycolytic flux to establish immunosuppressive niches (Kao et al., 2022), whereas PD-1/PD-L1/CTLA-4 blockade counteracts this by metabolically reprogramming TILs to restore effector functions (Zappasodi et al., 2021; Parry et al., 2005; Patsoukis et al., 2015; Qorraj et al., 2017). Crucially, glucose availability directly regulates PD-L1 expression to promote immune escape (Guo et al., 2022), while glucose restriction induces p53-dependent apoptosis that amplifies antitumor immunity through antigen release (Wu Y. Q. et al., 2023). Spatial metabolomic platforms (e.g., MALDI-MSI) provide critical insights into combination therapy dynamics via *in situ* metabolic mapping. Applied to A549 spheroid models, this approach revealed treatment-induced spatial metabolic heterogeneity (e.g., compartmentalized lipid alterations) and established quantitative correlations between drug penetration depth and metabolite gradients (Chen et al., 2021) -informing rational design of combinatorial schedules.

Research reveals that heightened glutaminolysis in tumor cells competitively depletes metabolic resources, profoundly suppressing T cell functionality. Targeting this pathway induces metabolic stress in malignant cells, reducing glutamine-to-glutamate conversion and triggering redox imbalance coupled with mitochondrial stress. Concurrently, it reprograms T cell metabolism to enhance survival, proliferation, and effector responses. Critically, glutaminase inhibition differentially modulates metabolic states across T cell subsets to potentiate antitumor immunity (Lukey et al., 2017; Alkan et al., 2018; Araujo et al., 2017; Johnson et al., 2018). As the most abundant amino acid in tumor metabolism, glutamine targeting synergizes with checkpoint inhibitors (e.g., anti-PD-1) by simultaneously restricting tumor bioenergetics and enhancing T cell glycolytic flux, epigenetic reprogramming, and cytotoxicity (Leone et al., 2019; Su et al., 2020). This dual-action strategy holds clinical promise; rationally designed delivery systems could balance metabolic suppression in tumors with metabolic support in immune cells, opening new therapeutic avenues for ovarian cancer.

Lipid metabolic reprogramming of TAMs polarization represents a pivotal frontier for combination immunotherapy. Distinct from carbohydrate/amino acid interventions, lipid pathway modulation alters TME lipid synthesis/signaling to fundamentally redirect TAM polarization. TAMs chronically internalize lipids via CD36-mediated scavenging, inducing intracellular lipid accumulation. This metabolic rewiring activates fatty acid oxidation- OXPHOS axes to drive mitochondrial reprogramming, ultimately polarizing TAMs toward protumoral M2 phenotypes through STAT6-dependent signaling (Su et al., 2020). Enhanced intracellular lipid peroxidation and membrane lipid efflux further upregulate M2-associated genes while suppressing M1 transcripts (Goossens et al., 2019; Wu et al., 2019). Spatial omics resolves lipid metabolism-TAM polarization heterogeneity through high-resolution mapping of lipid distributions, metabolic enzyme expression, and TAM subpopulation topography. This identifies M2-TAM clusters spatially correlated with dysregulated lipid pathways, informing targeted delivery of lipid-modulating agents combined with checkpoint blockade in ovarian cancer (Nasir et al., 2023).

In summary, the convergence of glycolysis, glutamine, and lipid metabolic axes in ovarian cancer underscores a multifaceted immunosuppressive network amenable to integrated therapeutic intervention. Glycolysis inhibition disrupts tumor bioenergetics and PD-L1-driven evasion, restoring T cell cytotoxicity; glutamine blockade induces tumor redox stress while enhancing T cell metabolic fitness and effector functions; and lipid modulation reprograms TAM polarization from M2 to M1 phenotypes, alleviating protumoral signaling. These pathways intersect through shared mechanisms, such as nutrient competition, mitochondrial dysfunction, and spatial metabolic gradients, which collectively sustain TME immunosuppression. Unified strategies leveraging multi-axis inhibitors (e.g., pan-metabolic agents) combined with checkpoint blockade could synergistically amplify antitumor immunity, as evidenced by preclinical models showing enhanced efficacy when addressing cross-talk between these circuits. Spatial metabolomics further enables precision mapping of these interactions, guiding personalized combinatorial regimens to overcome resistance and improve clinical outcomes in ovarian cancer.

Although preclinical studies have demonstrated substantial potential for targeting tumor metabolism, most metabolism-targeted therapies have failed to meet their primary endpoints in clinical trials, and the underlying causes warrant in-depth reflection. The high heterogeneity and plasticity of tumor metabolism represent major obstacles. Different regions within the same tumor, or even distinct cell subpopulations, may adopt divergent metabolic pathways; this redundancy renders single-pathway inhibition prone to compensatory mechanisms. For instance, inhibition of glycolysis may trigger a compensatory increase in OXPHOS, while blocking glutamine metabolism can activate autophagy or alternative amino acid metabolic pathways. Second, the lack of effective patient stratification biomarkers poses a significant challenge. Most current trials enroll patients who have failed multiple lines of treatment, without screening based on the specific metabolic dependencies of their tumors. Ovarian cancer patients with different molecular subtypes or distinct TME backgrounds may exhibit vastly different responses to the same metabolic intervention. Third, the dual roles of metabolic pathways are often overlooked. Many key metabolites (e.g., lactate, ketone bodies) not only support tumor growth but also play crucial roles in regulating immune cell function. Unselective blocking of these pathways may simultaneously inhibit anti-tumor immunity, thereby attenuating therapeutic efficacy. Additionally, drug delivery barriers cannot be ignored. Abnormal blood supply and interstitial hypertension within tumors may limit the accumulation of drugs in metabolically active hypoxic regions, leading to insufficient target inhibition. These failure experiences suggest that future metabolism-targeted therapies require more precise strategies: identifying specific metabolic dependencies using spatial multi-omics; developing reliable patient stratification biomarkers; and rationally designing combination therapy regimens that account for the integrated effects of metabolic interventions on both tumor cells and the immune microenvironment.

### 5.2 Development of metabolism-immunity dual-targeting agents

The metabolic-immune interplay within tumor microenvironments offers novel paradigms for bispecific therapeutic development. Integrating spatial metabolomics to resolve metabolic heterogeneity with pharmacokinetic modeling, organoid validation platforms, and multi-omics analytics provides a robust framework for overcoming immunotherapy resistance.

Spatially resolved metabolic landscapes enable molecular stratification for dual-targeting therapies. MALDI-MSI profiling reveals immunosuppressive niche-specific accumulation of lactate, kynurenine, and glutamine. Metabolic subtype-guided interventions include: LDHA inhibitors combined with PD-L1 blockade for glycolytic-dominant tumors (Ju et al., 2022); OXPHOS inhibitors targeting mitochondrial complex I for OXPHOS-dependent subtypes (He et al., 2023); FASN inhibitors with anti-PD-L1 for lipid-metabolism dysregulated cancers (Huang et al., 2024); and dual IDO1/TDO blockade for amino acid-addicted malignancies (Liang et al., 2021).

Precision intervention requires optimization of spatiotemporal drug distribution. Clinical trials comparing rifampicin versus rifapentine in cavitary tuberculosis revealed no superiority despite rifapentine’s lower MIC in murine models. MALDI-MSI-guided PK/PD modeling identified differential cavity penetration kinetics (Rifat et al., 2018), demonstrating how spatial metabolomics can optimize drug scheduling for ovarian cancer combination regimens.

Lactate metabolism orchestrates dual immunoregulatory functions. Tregs exploit lactate to maintain immunosuppression through CTLA-4 upregulation via RNA splicing modulation-a mechanism exploitable by CTLA-4 blockade (Ding et al., 2024); and it establishes acidic microenvironments that promote metastasis while impairing antitumor immunity (Rizwan et al., 2013; Brand et al., 2016). Targeting lactate shuttling (e.g., MCT4 inhibition) with cisplatin demonstrates synergistic therapeutic potential (Chen et al., 2023).

Three-dimensional organoid platforms represent transformative tools for validating metabolic-immune target synergy. As innovative 3D culture systems, organoids uniquely preserve primary tumor heterogeneity and critical signaling pathways, enabling faithful recapitulation of *in vivo* drug response microenvironments. Patient-derived organoids coupled with high-throughput screening platforms provide robust validation of multi-target efficacy (Roy et al., 2017; Rauth et al., 2021). Integrated spatial transcriptomic-metabolomic analysis quantitatively resolves spatiotemporal drug penetration gradients and pharmacodynamic effects within organoid subregions (Zhou et al., 2025), offering novel solutions to therapy resistance arising from ovarian cancer heterogeneity.

The discovery of novel metabolic immune checkpoints heralds a new era for immunometabolic reprogramming. Translational research focuses on metabolism-enzyme-mediated immunomodulation and clinical intervention potential. Tumor-derived IL4I1 catabolizes tryptophan, activating the aryl hydrocarbon receptor (AhR) to enhance invasiveness and suppress antitumor immunity (Sadik et al., 2020). Both malignant cells and cDC1s overexpress glutamine transporter SLC38A2, facilitating glutamine uptake. Glutamine supplementation enhances cDC1-mediated antitumor responses, potentiating checkpoint blockade or adoptive T cell therapy (Guo et al., 2023). Glutaminolysis inhibitors reverse immunosuppression while directly activating T cells (Leone et al., 2019). Tumor-secreted lyophosphatidylserine suppresses ILC1 antitumor activity via GPR34 receptor, whereas GPR34 antagonism triggers potent ILC1-mediated tumor suppression (Yan et al., 2024). Future work will decode molecular networks linking metabolic reprogramming to immune evasion, leveraging single-cell metabolic flux analysis to design next-generation inhibitors that improve ovarian cancer response rates.

Spatial metabolomic stratification, organoid validation, and novel checkpoint discovery collectively establish an integrated framework from mechanistic insight to clinical translation (Table 2). Advancing this paradigm requires incorporating single-cell metabolic flux analysis and AI predictive modeling to optimize combinatorial sequencing, ultimately enabling precision dual-targeting immunotherapy.

**TABLE 2:** Clinical trial designs for metabolism-immunity guided precision therapy.

Target/Pathway	Therapeutic agents	Biomarker/Patient selection	Phase	Cancer type(s)	ClinicalTrials.gov identifier
IDO1	IDO1 inhibitor (Indoximod/1-D-MT) + anti-PD-1/PD-L1 (Pembrolizumab and Nivolumab)	Kynurenine/Tryptophan ratio	II/III	Metastatic melanoma	NCT03301636
BRAF + MEK	BRAF inhibitor (Vemurafenib) + MEK inhibitor (Cobimetinib) + anti-CTLA-4/PD-1 (Ipilimumab and Nivolumab)	Serum LDH level	Phase II	B-RAF V600 E/K mutant melanoma	NCT02968303
mTOR	mTOR inhibitor (ABI-009) + anti-PD-1 (Nivolumab)	PI3K/AKT1/MTOR pathway activity	I/II	NSCLC, SCLC, urothelial carcinoma, melanoma, RCC, HNSCC, HCC, etc.	NCT03190174
GLS1	GLS1 inhibitor (CB-839) + anti-PD-1 (Nivolumab)	Glutamine level	I/II	Clear cell renal carcinoma, melanoma, NSCLC	NCT02771626
FASN	FASN inhibitor (TVB-2640) + anti-HER2 (Trastuzumab)	Estrogen level	Phase II	HER2-positive metastatic breast cancer	NCT03179904
Tyrosine kinase receptor	Tyrosine kinase receptor inhibitor (Sitravatinib) + anti-PD-1 (Nivolumab)	-	I/II	Metastatic renal cell carcinoma	NCT03015740

Table 2 summarizes representative clinical trials that illustrate the rationale for co-targeting metabolic pathways and immunotherapy. Notably, as of the writing of this review, the clinical translation of this strategy remains in its early stages, with a marked paucity of trials specifically targeting ovarian cancer. This gap may reflect long-standing challenges in the field of metabolic targeting, such as achieving tumor specificity to avoid on-target systemic toxicity. The trials listed herein, primarily focusing on melanoma, renal cell carcinoma, and non-small cell lung cancer, serve as pioneering “proof-of-concept” studies. Their design-highlighting specific metabolic targets (e.g., IDO1, GLS1), rational combination partners (e.g., anti-PD-1), and corresponding biomarker strategies-provides a critical translational roadmap and source of testable hypotheses for future ovarian cancer-specific clinical research.

### 5.3 Spatial omics-guided precision therapeutics

Spatial omics-driven personalized therapy overcomes limitations of conventional approaches by resolving spatiotemporal heterogeneity within TME. Mapping regionalized metabolic-immune networks enables spatiotemporally optimized interventions through stimuli-responsive nanocarriers and AI-powered predictive modeling (Dergaa et al., 2024).

Immunotherapy resistance stems fundamentally from spatially driven immune evasion. Despite clinical successes, most patients fail to achieve durable responses. Cytotoxic T lymphocytes (CTLs) eliminate tumor cells via TCR-pMHC recognition followed by perforin/granzyme-mediated apoptosis. Cancers evade this through antigen presentation downregulation (Zaretsky et al., 2016; Sade-Feldman et al., 2017). Glycolysis/Glut1 inhibition enhances CTL cytotoxicity, revealing novel immunotherapeutic targets (Wu et al., 2023b). Spatial heterogeneity critically drives therapeutic resistance, as evidenced by DCE-MRI radiomics predicting treatment response in triple-negative breast cancer (Ramtohul et al., 2024)-a paradigm applicable to ovarian cancer decision-making.

Precision targeting requires intelligent localized delivery systems. Tumor-responsive nanoparticles exploit microenvironmental cues (e.g., disulfide cleavage) for site-specific drug release (Yan et al., 2020; Duan et al., 2017; Zhu et al., 2024). Externally activated systems (light/ultrasound/magnetism/X-ray) trigger payload deployment at irradiated sites (Shen et al., 2017; Zheng et al., 2024). Accumulated nanotherapeutics release agents via passive/active targeting upon external stimulation (Chu et al., 2019; He et al., 2018; Chen et al., 2017), while multistage nanoparticles enable synergistic ROS generation/pH modulation/drug release under combinatorial stimuli (Liu et al., 2017; Sun et al., 2019; Zhao et al., 2020).

Artificial intelligence integrates multimodal spatial data for clinical decision support. Machine learning algorithms decipher nonlinear, high-dimensional relationships across genomics, epigenomics, transcriptomics, proteomics, metabolomics, and radiomics (Esteva et al., 2019; Bera et al., 2019; Bhinder et al., 2021), enabling early detection, molecular subtyping, and treatment response monitoring (Cohen et al., 2018; Capper et al., 2018; Shimizu and Nakayama, 2019; Xu et al., 2022).

**FIGURE 6:**
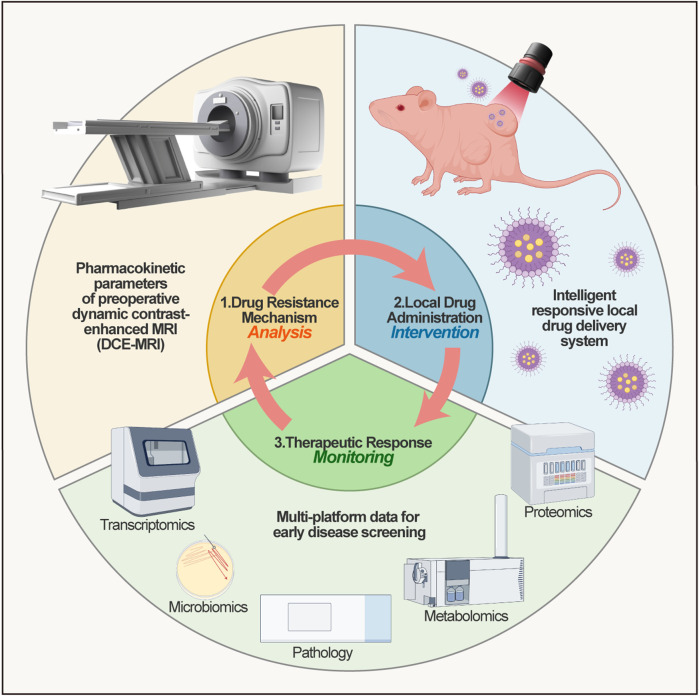
Precision Therapeutic Closed Loop Driven by Spatial Multi-Omics. The process begins with preoperative dynamic contrast-enhanced MRI (DCE-MRI) to characterize intratumoral pharmacokinetic heterogeneity, identifying resistant regions and guiding localized drug delivery. This is followed by intelligent drug administration intervention, utilizing a smart responsive localized drug delivery system to monitor therapeutic response. Finally, multi-platform data from transcriptomics, microbiomics, pathomics, proteomics, and metabolomics are integrated for real-time efficacy monitoring and algorithm iterative updates, forming a continuously optimized precision therapeutic closed loop. Created by Figdraw.

Converging nanotechnology with AI-driven modeling establishes an end-to-end framework from mechanistic insight to clinical translation (Figure 6), offering interdisciplinary solutions for spatial precision medicine in treatment-refractory ovarian cancer.

## 6 Future directions and challenges

Spatial multi-omics technologies, while revolutionizing our understanding of ovarian cancer’s metabolism-immunity axis, present persistent challenges alongside transformative opportunities. Future advances must overcome technical limitations, deepen mechanistic insights, and accelerate clinical translation to realize precision immunometabolic therapeutics.

At the forefront of technology, the development of imaging techniques that can non-invasively capture, in real time, dynamic metabolic processes and immune cell activities within living biological systems is a pivotal breakthrough for deciphering the spatiotemporal dynamics of metabolic-immune interactions. *In vivo* imaging employs radiographic approaches at the cellular and molecular levels in living organisms, allowing qualitative and quantitative assessment of biological processes and their changes over time. The marked heterogeneity of the tumor metabolic microenvironment severely constrains the effectiveness of metabolism-targeted therapeutics. By applying an *in vivo* dynamic imaging technique that uses blood oxygen saturation as a marker, it is feasible to clearly visualize tumor vascular structures in deep mouse tissues while quantifying SO_2_ levels, thus evaluating tumor metabolic activity and predicting responses to cancer immunotherapy (Fang et al., 2024). Observing molecular signaling changes within tumor cells via *in vivo* optical molecular imaging to explore mechanisms that facilitate intracellular antigen release and binding to circulating antibodies will aid in advancing the development and application of intracellular antigen-based tumor vaccines (Dai et al., 2022). Using adaptive scanning light-field microscopy (DAOSLIMIT), the movement paths of neutrophils and monocyte-macrophages in mouse livers during acute AILI have been vividly depicted with ultra-high spatiotemporal resolution; when combined with single-cell multi-omics, this has uncovered the immunometabolic landscape of drug-induced liver injury (Chen et al., 2024). The most recent *in vivo* deep-tissue dynamic metabolic and structural imaging assay leverages an MMF excitation source to power the enhanced dynamic capture of dSLAM microscopy, enabling *in vitro* tracking of monocyte behaviors and simulation of immune cell recruitment within living vascular networks (Liu K. et al., 2024).

Beyond the tumor’s own microenvironment, the gut microbiota and their metabolites-acting as key systemic modulators-demonstrate significant potential in shaping the tumor immune microenvironment and affecting immunotherapy outcomes, making them a crucial focus for future interventions. The gut microbiota remodels the TME by regulating innate and adaptive immune cell functions, exerting a notable impact on the clinical success of immunotherapy (Jiang et al., 2025). Their metabolites, including short-chain fatty acids, inosine, trimethylamine-N-oxide, and succinate, play key roles in modulating immune responses (Mager et al., 2020; Nomura et al., 2020; Wang H. et al., 2022; Wu et al., 2020). Intratumoral microbiota enhance anti-tumor immunity through mechanisms such as STING pathway activation (Shi et al., 2020), while the gut microbiota not only dictates treatment efficacy but also is closely linked to immune-related adverse events (irAEs) like ICI-associated colitis (Tandon et al., 2018; El Osta et al., 2017), modulating gut microbiota composition via strategies such as fecal microbiota transplantation (FMT), dietary adjustments, and probiotic supplementation (Matson et al., 2018; Goncalves et al., 2020; Huang et al., 2022) provides new therapeutic directions to improve ovarian cancer treatment outcomes and mitigate immune toxicity.

Ultimately, addressing the core challenges in life sciences-limitations of single-omics explanations, obstacles to multi-omics integration, and gaps in clinical translation-relies on systematic innovation and collaboration across technologies, methods, and frameworks. The integration of spatial omics and single-cell sequencing is redefining research frontiers. Advances in data analysis are dismantling dimensional barriers. Refining clinical translation pathways demands bidirectional validation systems. Multi-omics research is trending in three areas: *in situ* detection technologies overcoming spatiotemporal resolution constraints, AI-driven knowledge graphs integrating public databases, and the pairing of miniaturized detectors with cloud-based analysis platforms to lower research costs. Going forward, developing standardized analytical pipelines, constructing clinical sample repositories and multi-omics databases, and creating modular experimental designs are all valuable goals.
